# Importance of the One Health approach to study the SARS-CoV-2 in Latin America

**DOI:** 10.1016/j.onehlt.2020.100147

**Published:** 2020-06-25

**Authors:** D. Katterine Bonilla-Aldana, Yeimer Holguin-Rivera, Soffia Perez-Vargas, Adrian E. Trejos-Mendoza, Graciela J. Balbin-Ramon, Kuldeep Dhama, Paola Barato, Charlene Lujan-Vega, Alfonso J. Rodriguez-Morales

**Affiliations:** a Semillero de Investigacion en Zoonosis (SIZOO), Grupo de Investigacion BIOECOS, Fundación Universitaria Autónoma de las Américas, Sede Pereira, Pereira, Risaralda, Colombia; b Public Health and Infection Research Group and Incubator, Faculty of Health Sciences, Universidad Tecnológica de Pereira, Pereira, Risaralda, Colombia; c Master in Clinical Epidemiology and Biostatistics, Universidad Cientifica del Sur, Lima, Peru; d Hospital de Emergencias Jose Casimiro Ulloa, Lima, Peru; e Division of Pathology, ICAR-Indian Veterinary Research Institute, Izatnagar 243 122, Bareilly, Uttar Pradesh, India; f Corporación Patología Veterinaria (Corpavet), MolecularVet SAS, Bogotá, Colombia; g Avi-Vet Servicios, Lima, Peru; h Department of Anatomy, Physiology & Cell Biology, School of Veterinary Medicine, University of California, Davis, California, United States; i Grupo de Investigación Biomedicina, Faculty of Medicine, Fundación Universitaria Autónoma de las Américas, Pereira, Risaralda, Colombia; j Universidad Privada Franz Tamayo, Cochabamba, Bolivia

**Keywords:** One health, SARS-CoV-2, COVID-19, Spillover, Zoonotic

## 1 Introduction

The Coronavirus Disease 2019 (COVID-19) pandemic, due to the severe acute respiratory syndrome coronavirus 2 (SARS-CoV-2) is continuing and is currently raging in Latin America [[1], [2], [3], [4]]. In this region (including the Caribbean), up to July 3, 2020, 2,746,277 cases have been reported, more than 2,358,756 of them in South America, and 1,496,858 just in Brazil [5,6]. This, the largest country in the region, have reported 61,884 deaths (4.13%). Mexico, the second largest country, has a higher proportion of deaths, 29,189 out of 238,511 (12.23%). On the other side, Chile, although have reported 288,089 cases, only 6,051 deaths have registered (2.1%) [5,6]. In the case of Venezuela, this a country where doubts about the numbers have been raised. Up to July 3, 2020, 6,273 cases have been reported, but, this number could be underestimated because of under-testing and under-reporting [7]. In the region, there have been clear differences in the responses to the disease, with countries such as Colombia, Peru, Bolivia, Chile, Argentina, among others following wide recommendations of quarantine, physical distance and biosecurity education in an early stage of the pandemic, whilst in others such as Brazil or Mexico, this has been delayed, with very well-known consequences. In Brazil, the poorly-urbanized neighborhoods on the margins of city centers, the so called favelas, are focal points for the disease, with precarious living conditions and high population density making social distancing a near-impossibility [8].

The coronaviruses (CoVs) are pathogens that can be transmitted between and infecting in both humans and animals. These have a worldwide distribution [[9], [10], [11], [12], [13]]. Due to the importance of the SARS-CoV-2 outbreak that started in Wuhan, province of Hubei, China, the World Health Organization (WHO) declared this viral infection as a health emergency of international concern and later as a pandemic [[14], [15], [16], [17], [18], [19], [20]]. Taxonomically, this virus is an enveloped single-stranded RNA virus, which belongs to the subgenus *Sarbecovirus*, part of the genus *Betacoronavirus* (order Nidovirales; suborder Cornidovirineae; family Coronaviridae; subfamily Coronavirinae). In the subgenus *Sarbecovirus*, is also included the SARS-CoV, the etiological agent of the 2002–2003 epidemic originated in Guandong, China [[21], [22], [23]]. For One Health, viruses among the Coronaviridae family are archetypal [24]. The virus shares a high level of identity with some bat coronaviruses and is recognized as a zoonotic virus [24,25].

The virological and epidemiological scenario of this pandemic is complex, and still, many questions remain unanswered. Animal infection due to zoonotic coronaviruses has been previously reported on a farm and domestic animals such as cattle, pigs, dogs, among others [26]. This probably have been occurring for years, and not only in animals but in asymptomatic humans. Nevertheless, the first significant and apparent report in humans was described in 2002 in Guangzhou, province of Guangdong, China, where more than eight thousand cases were confirmed due to a new virus, causing 774 deaths in 32 countries around the world [27]. That virus was the SARS-CoV [[27], [28], [29], [30], [31]]. Later in 2012, another outbreak of a zoonotic coronavirus was reported. The Middle East Respiratory Syndrome coronavirus (MERS-CoV), originated in Saudi Arabia, also spread to other Asian, African, European, and American countries, also causing deaths [11,[32], [33], [34]].

**Fig. 1:**
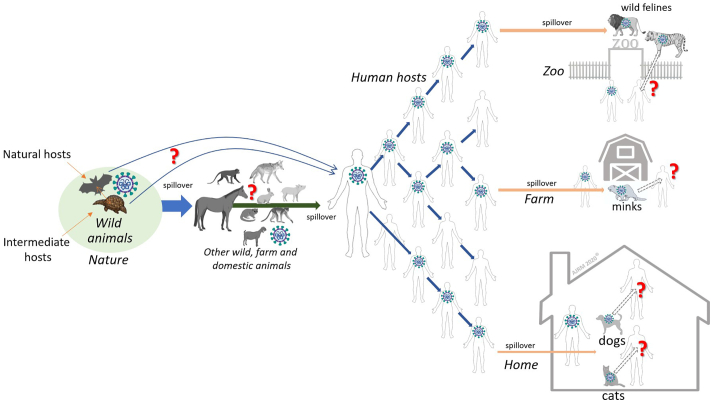
Proposed current evidence-based interactions between animal, human, and environmental components in the context of SARS-CoV-2 transmission.

In the case of the SARS-CoV, it was shown that the outbreak originated due to the transmission of the Himalayan civet (*Paguma larvata*) [30]. However, it was also reported that animal species such as raccoons and bats could carry the virus [30]. In the case of MERS-CoV, after identifying the virus, the epidemiological relationship between human and camel cases was confirmed [[34], [35], [36]]. Both SARS-CoV and MERS-CoV have caused more than 10,000 cumulative cases in the past two decades, with case fatality rates of 10% and 37%, respectively [[34], [35], [36]]. The identified coronaviruses were the tip of the iceberg. More of them would emerge and become apparent as already occurred with the current pandemic SARS-CoV-2/COVID-19 [11,12,23]. In this scenario of interaction between animal and human health, also the environmental health, then, the concept of One Health, is again of utmost importance. One Health is an approach that recognizes that the health of people is closely connected to the health of animals and our shared environment (Fig. 1). Currently at homes, zoological parks and farms, there have been reports of infections from humans to animals. At homes, cats and dogs; at zoological parks, tigers and lions; and at farms, minks (Fig. 1) [[37], [38], [39], [40]].

At the integrative disease ecology study of SARS-CoV-2/COVID-19, its relevance seems to be more than evident. Notably, at the beginning of the pandemic, during the first identified spillover between animals and humans, at the Huanan Seafood Wholesale Market in Wuhan or in the suburban-wild interphases or ecotones that probably allow the interaction between SARS-CoV-2 infected animals and humans, this should be a relevant factor (Fig. 1). Current evidence is showing that the SARS-CoV-2 would be transmitted from humans to domestic cats at home, as well as to wild felids in zoological parks (Fig. 1), without evidence of transmission from these animals to humans [26,37,[41], [42], [43], [44]].

## 2 Animals

Nevertheless, at this moment we do not know the real susceptibility of animals to SARS-CoV-2 exposure, including the role of the inoculum, the route of transmission, among other related factors. In the current scenario of significant human-to-human transmission appear as the main focus to be attended, and the role of animals would be seen as marginal, but this deserves a comprehensive approach and assessment to understand the epidemiology and future advance of disease. If individual animals became reservoirs or vehicles of SARS-CoV-2, this infection might become endemic. In the case of South America, as yet, so not reports of transmission from human to animals have been made, nevertheless, in addition to dogs, cats, tigers and lions, maybe there is concern about infection in *Mustelidae* from South America [45], but this is yet to be seen and reported.

## 3 Environment and degradation

In the context of COVID-19 pandemic, the presence of SARS-CoV-2 in wastewater has been consider as a potential health risk, but also as an effective approach to predict the potential spread of the infection by testing for infectious agents in wastewater [[46], [47], [48], [49]]. Given that plumbed wastewater is not universal and in places such as suburban areas of cities in northeastern Brazil, a lot of human waste is pumped directly into lakes and streams. Recently, more studies are showing the detection of SARS-CoV-2 in wastewater [[50], [51], [52], [53], [54], [55], [56], [57], [58], [59], [60], [61]]. In other countries of South America the situation is even more complicated as there are not enough wastewater treatment plants with its consequent implications, including the SARS-CoV-2 context.

In the environmental context, the impact of climate change and land use, including deforestation and intensive farming practices, should also be analyzed [62]. Disruptions in environmental conditions and habitats can provide new opportunities for SARS-CoV-2 and maybe other CoVs to spillover. Given the great abundance of non-human primates in South America, especially in Rio de Janeiro, where tourists are in close contact,  with callitrichid monkeys, and there is recent evidence indicating that rhesus macaques are susceptible to SARS-CoV-2 [63]. Human cases in these niches are a matter of concern. Species of mustelids, canids and felids from the order Carnivora are known to be SARS-CoV-2 positive. In Iguazu, Argentina, tourists are in close contact with coatis, a species of procyonid from the order Carnivora [64]. Although at this moment there is no evidence of any procyonid species host SARS-CoV-2, close contact with humans could be an issue in this region.

In the case of Latin America such disruptions have been seen especially in the Amazon jungle, an area shared not only with Brazil, but with many other countries in South America, observing the impact on zoonotic and vector-borne diseases and pathogens, such as malaria, dengue, chikungunya, Zika, hantavirus, hemorrhagic viral fevers [62,[65], [66], [67]].

## 4 Conclusions

Health programs targeting an integrative approach for COVID-19 should consider the role of One Health initiatives [9,68]. Operative research on animals in close contact with positive SARS-CoV-2 humans, beginning with those at home or in zoological parks, such as dogs, pets, ferrets among others, should be studied, as recent evidence suggests the possible human-to-felines and human-to-dogs transmission (Fig. 1) [[69], [70], [71], [72]]. Many unanswered questions need to be carefully studied with ONE HEALTH approach [73]. This would be helpful for a better understanding of SARS-CoV-2/COVID-19 epidemiology, transmission, dynamics, and disease ecology. In Colombia, associations such as the Colombian Association of Veterinarians Attending Small Animals (VEPA), are promoting discussions around SARS-CoV-2 and the importance of One Health, to inform the society about the evidences regarding transmission in some domestic animals, felines, including cats, lions, and tigers, as well as to promote the development of expert panels; also, these discussions including the evaluation of capacities of veterinary molecular laboratories in case that could be needed support to SARS-CoV-2 diagnosis in humans [74]. In Peru, the College of Veterinary Medicine, is developing statements and providing recommendations especially for the health authorities. In Chile, the Chilean Society of Zoonoses has been working in wide diffusion of information and education to population regarding human and animal implications of the SARS-CoV-2/COVID-19 pandemic. In Brazil, the Brazilian Society of Zoology is developing online training courses, conferences and symposia directly targeted to the One Health approach in the context of the SARS-CoV-2/COVID-19 pandemic. Unfortunate, in countries such as Venezuela, there is a lack of responses directly targeting these approaches. Furthermore, multiple teams composed by engineers, biologists, physicists, veterinarians, public health professionals are making efforts to develop Colombian and Peruvian products such as mechanical ventilators, N95 masks, and ozone cabinets to aid to contain the effects of this disease. However, not in all cases there is an integration of combining the knowledge from a multidisciplinary and multi-institutional team.

Health is one. Now is time to make this critical message to deliver to health authorities and society. The study and control of all emerging and zoonotic diseases require this approach. It will benefit the understanding and the opportunities to deploy early interventions and mitigate the profound impacts, a pandemic of zoonotic origin has.
